# miRNAs in the Pathogenesis of Systemic Lupus Erythematosus

**DOI:** 10.3390/ijms16059557

**Published:** 2015-04-28

**Authors:** Bo Qu, Nan Shen

**Affiliations:** 1 Department of Rheumatology, Renji Hospital, School of Medicine, Shanghai Jiao Tong University, Shanghai 200001, China; E-Mail: qubo.bio@gmail.com; 2 Institute of Health Sciences, Shanghai Institutes for Biological Sciences (SIBS) & Shanghai Jiao Tong University School of Medicine (SJTUSM), Chinese Academy of Sciences (CAS), Shanghai 200031, China; 3 Division of Rheumatology and the Center for Autoimmune Genomics and Etiology (CAGE), Cincinnati Children’s Hospital Medical Center, Cincinnati, OH 45229-3039, USA

**Keywords:** microRNA, systemic lupus erythematosus, innate immunity, adaptive immunity, lupus nephritis, biomarker, therapy

## Abstract

MicroRNAs (miRNAs) were first discovered as regulatory RNAs that controlled the timing of the larval development of *Caenorhabditis elegans*. Since then, nearly 30,000 mature miRNA products have been found in many species, including plants, warms, flies and mammals. Currently, miRNAs are well established as endogenous small (~22 nt) noncoding RNAs, which have functions in regulating mRNA stability and translation. Owing to intensive investigations during the last decade, miRNAs were found to play essential roles in regulating many physiological and pathological processes. Systemic lupus erythematosus (SLE) is a chronic autoimmune disease characterized by elevated autoantibodies against nuclear antigens and excessive inflammatory responses affecting multiple organs. Although efforts were taken and theories were produced to elucidate the pathogenesis of SLE, we still lack sufficient knowledge about the disease for developing effective therapies for lupus patients. Recent advances indicate that miRNAs are involved in the development of SLE, which gives us new insights into the pathogenesis of SLE and might lead to the finding of new therapeutic targets. Here, we will review recent discoveries about how miRNAs are involved in the pathogenesis of SLE and how it can promote the development of new therapy.

## 1. The Biology of miRNA

miRNA genes are mainly located within intronic regions of a genome, with a few in exonic regions [1]. miRNAs tend to be encoded in a clustered manner, such that nearly 50% of mammalian miRNA loci are found adjacent to other miRNAs [2]. Clustered miRNAs can either be transcribed as one primary transcript and regulated by a common promoter or transcribed individually using different promoters [3]. Although a few miRNAs associated with Alu repeats can be transcribed by RNA polymerase III (Pol III) [4], Pol II is used to transcribe most miRNA genes [5]. After being transcribed, primary transcripts of an miRNA gene (pri-miRNAs) are subjected to cleavage by Drosha in the nucleus [6], which is the first step of the miRNA maturation process. Subsequently, a small hairpin RNA (pre-miRNA) is released and exported to the cytoplasm by Exportin-5 [7]. In the cytoplasm, pre-miRNAs are the substrates of another RNase III, Dicer. With the help of other double-stranded RNA-binding domain (dsRBD) proteins, Dicer dices the pre-miRNA into a ~22 nt-long duplex RNA, which contains two mature miRNA strands [8]. Based on their thermodynamic properties, one of the two strands will be preferred to incorporate into the RNA-induced silencing complex (RISC) that contains the Argonaute (Ago) family protein that is responsible for exerting the inhibitory function of the miRNA [9]. The other strand, which is called miRNA* and exists at a very low level, was considered to have no function and finally to be degraded by the cell. However, more and more evidence indicates that these miRNA* strands can indeed accumulate to a certain level and are functional in some cases [10,11]. (Therefore, the use of the miR/miR* nomenclature is ceased, and instead, -5p or -3p was used for the sequences derived from the 5' or 3' arms of the hairpin precursor [12]. However, we will still use the old nomenclature in order to be consistent with the original research paper).

miRNAs are mainly regulated at the transcription level, which gives them a cell type-specific and spatiotemporal expression pattern [13]. Many characteristics of miRNA gene promoters are similar to those of protein coding genes, and thus, miRNAs can be regulated by the mechanisms that are found to take part in regulating protein coding genes, such as the binding of transcription factors or enhancers to the cis regulatory elements, DNA methylation or histone modification status of the promoter [14]. Moreover, the expression of miRNAs can also be regulated post-transcriptionally. In the nucleus, Drosha needs *DGCR8* (DiGeorge critical region 8) to facilitate pri-miRNA processing [15]; while several other accessory factors, such as *EWSR1*, *DDX5* and *DDX17*, can promote the fidelity and activity of Drosha processing [16]. In the cytoplasm, like Drosha, Dicer needs other dsRBD proteins, TRBP, to dice pre-miRNAs into the miRNA duplex [17]. Other proteins can also affect miRNA maturation by regulating these accessory components in miRNA processing. For example, *TRIM71* was found to regulate *let-7* biogenesis by ubiquitinating *Lin28B*, which regulated *let-7* maturation by recruiting TUT4 to block Dicer function [18,19].

By base-pairing of its seed region (nucleotides 2–8 from the 5' end of a miRNA) to the 3'UTR of a target mRNA or to the coding regions of a target mRNA, mature miRNAs mediate the binding of RISC to their targets, then inhibit the translation of the mRNAs and even cause degradation of the mRNAs [20]. Each mature miRNA has the ability to target multiple different mRNAs. On the contrary, a particular mRNA can be targeted by multiple miRNAs that regulate the expression of the same gene in concert.

Similar to miRNA biogenesis, many factors can affect miRNA’s binding to its target mRNA and its subsequent inhibitory function. As for miRNA itself, nucleotides in the middle of an miRNA could have influence on its targeting relationship with mRNAs [21]. RNA-binding proteins (RBPs), such as *DND1* and HuR, which bind to the 3'UTR of a mRNA, can interfere with RISC’s binding to its target and protect certain mRNAs from miRNA-mediated repression [22,23]. There is evidence that circular RNAs (circRNAs) serve as potential regulatory factors for both the level and function of miRNAs [24]. Ago proteins in RISC are one of the regulatory targets, which are used to control miRNA function. *TRIM71*, a member of the TRIM-NHL family proteins, can directly associate and ubiquitinate Ago2 protein and, thus, regulate its turnover [25].

By fine-tuning gene expression, miRNAs can function as key regulatory factors in many biological processes, including cell proliferation, cell differentiation, apoptosis, organogenesis, development and immune responses [26,27]. Abnormal expression or function of miRNAs has been found in many diseases, such as cancer, heart diseases and autoimmune diseases [28,29,30].

## 2. miRNAs in the Pathogenesis of SLE

It is interesting that autoimmune responses to some key components of the miRNA pathway were found in systemic lupus erythematosus (SLE), rheumatoid arthritis (RA) and Sjögren’s syndrome [30]. In SLE, it was reported that anti-Su autoantibodies, which were usually found in lupus patients, could recognize the catalytic enzyme in miRNA pathways (Ago1, Ago2, Ago3, Ago4 and Dicer) [31,32], which indicated the possible mutual relationship between miRNA and the pathogenesis of SLE. miRNA expression profiles using samples from a lupus mouse model and lupus patients revealed the clinical relevance of miRNAs in SLE [33,34]. Investigation of the function of those abnormally-expressed miRNAs sheds light on the mechanism of the development of SLE.

## 3. miRNAs Regulate Innate Immunity in SLE

Innate immune cells do not produce antigen-specific antibodies or receptors. However, by utilizing pattern recognition receptors (PRRs), innate immune cells can recognize dangerous signals derived from pathogens or damaged cells [35]. There are several PRRs families in the innate immune system: toll-like receptors (TLRs), NOD-like receptors (NLRs), RIG-I-Like receptors (RLRs), cytosolic DNA receptors (CDS); and ligand binding of these receptors triggers signaling cascades, which activate downstream transcriptional factors (such as NFκB and/or IRFs) and lead to the production of inflammatory cytokines and/or type I interferons [36,37,38]. These pro-inflammatory cytokines will further act in autocrine or paracrine manners to mediate the inflammation of local tissue, the recruitment and activation of many other immune cells and the initiation of the adaptive immune system [39].

The involvement of innate immunity in SLE pathogenesis has long been established [40]. The evidence that activation of innate receptor *TLR9* co-stimulated auto-reactive B-cells provided an explanation to epitope spreading in SLE [41]. Subsequent researches demonstrated that activation of *TLR9* accelerated renal disease in a lupus mouse model, MRL/MpJ-Fas^lpr^/J mice (common name MRL-lpr, mouse model that has spontaneous mutation of *FAS* gene and is prone to SLE) [42]. Type I interferon is recognized as one of the key pro-inflammatory cytokines in the pathogenesis of SLE [43]. Due to their key roles in stimulating downstream type I interferon production, TLRs, RLRs and CDSs on innate immune cells are recognized as important factors that participate in the initiation and augmentation of autoimmune responses in SLE [44,45]. *TLR7* was found to be essential for producing type I interferon and the development of lupus symptoms in an induced SLE model [46]. Consistently, translocation of *TLR7* to the Yaa locus accelerated systemic autoimmunity in another murine lupus model [47]. Many cytosolic DNA sensors could be induced by interferon and were found elevated in lupus patients [48]. Chronic activation of the STING-dependent cytosolic DNA sensing pathway may also be responsible for type I interferon production in SLE [49]. Several studies discovered that mutations in human *TREX1* were related to SLE [50]. *TREX1*-deficient mice showed elevated production of type I interferon and lupus-like symptoms, which was demonstrated to be mediated by STING-dependent pathways [51,52]. Tight regulation of the innate immune signaling pathway keeps our immune responses in control, protecting us from autoimmune diseases.

Several miRNAs were found to have crucial roles in negative regulating innate immune responses. Transcription of *miR-146a* could be induced by the engagement of several TLRs and pro-inflammatory cytokines, which stimulated downstream NFκB activity [53]. By targeting signaling adaptor proteins the TNF receptor-associated family (TRAF)-6 and IL-1 receptor-associated kinase (IRAK)-1, *miR-146a* suppresses NFκB activation and subsequent cytokine production [53]. *miR-146a* could also be able to inhibit type I interferon induction by *TLR7* and RIG-I pathway [54,55]. Thus, *miR-146a* can suppress type I interferon production by targeting multiple key molecules in the innate signaling pathway. Profiling of the miRNAs expressed in the PBMCs from lupus patients revealed that *miR-146a* was under-expressed in SLE [55]. Additionally, this was possibly due to a germline genetic variant in the *miR-146a* promoter [56]. Further investigation found that *STAT1* was another target of *miR-146a*, and there was a reverse correlation of *miR-146a* levels with the expression of interferon-inducible genes and SLE disease activity, indicating a critical role of *miR-146a* in excessive type I interferon production and signaling activity in SLE [55].

*miR-155* was another miRNA that has been investigated intensively for its function in regulating innate immune response. By targeting *MYD88* and *TAB2*, *miR-155* could inhibit inflammatory response [57,58]. On the contrary, *miR-155* promoted inflammatory response and type I interferon signaling by targeting the suppressor of cytokine signaling-1 (*SOCS-1*) in macrophage [59], which indicated the context-dependent character of the regulatory function of *miR-155*. One interesting discovery is that although originating from the same precursor, miR-155* has an opposite effect on the regulation of type I interferon production compared to that of *miR-155* in pDCs (plasmacytoid dendritic cells) [60]. When ligands bind to *TLR7* on pDCs, the transcription of the miR-155/miR-155* gene is activated, and this leads to rapid production of mature miR-155*, which increases the ratio of miR-155* to *miR-155*. Thus, miR-155* facilitates type I interferon production by inhibiting IRAKM expression. Type I interferon produced downstream of *TLR7* acts in an autocrine/paracrine manner to induce and activate KSRP, which will promote *miR-155* maturation, but not miR-155*. As *miR-155* accumulates, which inhibits *TAB2* in pDCs, the production of type I interferon and pDC activation are limited to a proper level. pDCs have been documented as the cells with great the ability to produce type I IFN when encountering nucleic acid, which could be recognized by the TLRs of pDCs. pDCs have been associated with SLE and might contribute to its pathogenesis by recognizing self-DNA or RNA, then producing a huge amount of pathogenic type I IFN [61]. Recent studies demonstrated that depletion of pDCs in BXSB/MpJ lupus-prone mice (males of this mouse model has mutant Yaa containing Y chromosome and develops severe form of SLE) ameliorated lupus pathology, which indicated that pDCs played critical roles during the IFN-dependent initiation of lupus and might be an attractive therapeutic target for treating SLE [62]. Considering the key role of type I interferon in SLE pathogenesis and pDCs’ strong capability of secreting type I interferon, further investigation of the expression of *miR-155* and miR-155* in pDCs in SLE will shed light on the mechanism of the deregulated type I interferon production.

Other miRNAs also play a part in regulating innate immune responses. *miR-3148* could target *TLR7* through binding to its 3'UTR, which provides us with an explanation of how a genetic variation found in the 3'UTR of *TLR7* mRNA affects its expression in SLE [63]. DCs from Blimp1-deficient mice showed increased expression of *let-7c*, which is responsible for the pro-inflammatory phenotype in DCs, and a similar regulatory mechanism has been found in human [64]. In addition, *let-7a*, the expression of which is elevated in the kidneys of SLE patients and in the mesangial cells of lupus mice, could promote E2F-mediated cell proliferation and the activation of NFκB [65].

## 4. miRNAs Regulate Adaptive Immunity in SLE

Due to the fact that autoantibodies are elevated in SLE, auto-reactive B-cells and T-cells are recognized to be important in disease pathogenesis and have been studied intensively and extensively [66,67]. Aberrant B-cell tolerance happens at multiple levels, which combines with deregulated BCR and BAFF signaling to promote the activation of auto-reactive B-cells [68,69]. Due to their ability to regulate B-cell responses, CD4+ T-cells were also found to have defects and to contribute to the abnormal adaptive immunity in SLE. CD4+ T-cells from SLE patients were demonstrated to have aberrant DNA hypomethylation, which provided an epigenetic explanation for excessive T-cell activation, but the mechanism was still unclear [70]. The miRNA profile of CD4+ T-cells from MRL-lpr mice identified *miR-21* and *miR-148a* as two upregulated miRNAs [71]. Further investigation found that *miR-21* and *miR-148a* could considerably reduce the *DNMT1* protein level, which was one of the major epigenetic components that had been linked to DNA hypomethylation in T-cells in SLE [72]. Additionally, *miR-21* inhibited RAS-MAPK-ERK signaling upstream of *DNMT1* in T-cells. Considering that *miR-21* was found to be upregulated in PBMCs in SLE patients and correlated with disease activity, it is possible that elevated *miR-21* and *miR-148a* contribute to the hypomethylation status of T-cells in SLE. Besides *miR-21* and *miR-148a*, *miR-126* was also reported to modulate DNA methylation in SLE CD4+ T-cells by directly targeting *DNMT1* [73], which provided us a more comprehensive explanation of DNA methylation in CD4+ T-cells in SLE. *miR-29b*, which is upregulated in SLE CD4^+^ T-cells, has been shown to inhibit *SP1* expression in human T-cells, meaning that they inhibit the expression of *DNMT1* and modulate DNA methylation [74], and further studies demonstrated that inhibition of *miR-29b* in the T-cells of SLE patients reversed DNA hypomethylation and the upregulation of downstream genes.

Inflammatory cytokines and chemokines are dysregulated and have crucial roles in the development of SLE. For example, the expression of *IL2* in T-cells from SLE patients is dramatically lower than that from healthy controls [75]. *IL2*, mainly functioning in a paracrine manner, was proposed to have an indispensable role of maintaining natural regulatory T-cells in the periphery [76] and, thus, could contribute to the uncontrolled immune responses. *CCL5* (or RANTES) is one of the many overexpressed chemokines in sera from lupus patients [77]. *CCL5* plays a critical role in regulating immune cell movement and is indicated to have functions in lupus-associated tissue damage [78]. In addition to the transcription regulators, such as *CREM* (cAMP-responsive element modulator), *CREB1* (CREM-binding protein 1) and NFAT (NFκB, AP1 and nuclear factor of activated T-cells), it was shown that *miR-31*, downregulated in SLE T-cells, could repress the expression of RhoA, a negative regulator of NFAT, and was responsible for impaired *IL2* expression [79,80]. Moreover, the expression of *miR-31* was inversely correlated with RhoA in SLE patients [80]. Another miRNA, *miR-125a*, was found significantly downregulated in PBMCs from SLE patients [81]. Because of its selective expression in T-cells and its target relationship with *KLF13*, *miR-125a* was suggested to promote the secretion of *CCL5* by SLE T-cells [81]. Thus, other than epigenetic traits, miRNAs were also involved in regulating cytokine production in T-cells in SLE.

The autoantibodies in lupus patients are high-affinity, somatically-mutated and Ig-switched, indicating that there were defects of germinal center (GC) responses in SLE [82]. Tfh cells (follicular helper T cells), derived from the differentiation of naive T-cells, mediate memory and plasma B-cells formation in GC (germinal center), and crosstalk between Tfh cells and B-cells will lead to class switching and affinity maturation of B-cells [83]. So far, studies of miRNAs in Tfh cell differentiation have been mainly focused on the miR-17~92 cluster. Mice that have the transgene of the miR-17~92 cluster developed lymphoproliferative disease and autoimmune symptoms, like SLE [84]. On the molecular level, miR-17~92 was shown to repress the expression of *PTEN* and *PHLPP2* and regulate Tfh cell differentiation and function [85,86]. As for B-cells, researchers found that elevated expression of *miR-30a* was responsible for the underexpression of *LYN* in SLE B-cells [87]. Additionally, more efforts are need to investigate miRNAs in B-cells.

## 5. miRNAs Regulate Inflammation in Tissue-Resident Cells in SLE

Usually, SLE patients have multiple clinical features and organ damage caused by inflammation. IL17 is one of the most studied pathogenic pro-inflammatory cytokines, and it has been shown to be overexpressed in target tissues and to be responsible for local inflammation in many autoimmune diseases, such as RA, multiple sclerosis and SLE [88,89,90]. *miR-23b* was found underexpressed in target tissues in a study that comprehensively profiled miRNA expression in affected tissues of patients with SLE or RA, as well as mouse models of SLE or RA [91]. Subsequent results showed that *miR-23b* could target *TAB2*, *TAB3* and IKK-α to inhibit IL17, TNF or IL1β signaling. These findings emphasized the regulatory role of miRNAs in tissue-resident cells. Lupus nephritis is one of the major complications associated with SLE [92]. Besides immune disorders, renal tissue also plays an important role in local inflammation. miRNA expression profiles of renal tissue, which identified 36 upregulated and 30 downregulated miRNAs in the renal tissues of lupus nephritis patients, provided a good source of differentially-expressed miRNAs in lupus kidney tissues [93]. Recent results showed that *let-7a* was significantly increased in mesangial cells of NZBWF1/J mice (common name NZB/W, this mouse model develops an autoimmune disease resembling human SLE) compared to age-matched NZW/LacJ mice (common name NZW, F1 hybrids of NZW/LacJ and NZB/BlNJ are used as a model for human SLE) and might be involved in regulating *IL6* production in these cells [65]. Other than regulating inflammation, miRNAs were found to mediate renal fibrosis in lupus nephritis. By comparing miRNA expression in kidney biopsies of lupus nephritis patients, researchers identified *miR-150* with a positive correlation with chronicity scores and the expression of profibrotic proteins [94]. Furthermore, they found that TGFβ1 could induce *miR-150*, and elevated *miR-150* could target antifibrotic protein *SOCS1* with upregulated profibrotic proteins in renal proximal tubular and mesangial cells. These findings suggested that TGFβ1 executed its profibrotic effects partly through *miR-150*. 

## 6. miRNAs as Biomarkers of SLE

Over the past few decades, tremendous efforts have been devoted to tackling the mechanisms for the initiation and development of the autoimmunity and subsequent multiple organ damage of SLE. Although significant progress has been made, there are still many other challenges to conquer the disease. One of those important needs is the lack of reliable biomarkers for diagnosis, monitoring, stratification and prediction of prognosis [95].

As the investigation of the functions of lupus-associated miRNAs goes on, it has been recognized that miRNAs are dysregulated in SLE [33,96,97]. Recent studies showed that there were distinct expression patterns of miRNAs in peripheral blood leukocytes of SLE patients. Additionally, these specific patterns were found to be associated with different autoantibody compositions in those SLE patients [98]. Another study identified aberrant expression of miRNAs (especially hsa-miR-371-5p, hsa-miR-423-5p, hsa-miR-638, hsa-miR-1224-3p and hsa-miR-663) in the PBMCs of lupus nephritis patients across different patients with different races [99]. With the discovery of the existence of miRNAs in body fluid, miRNAs are becoming an ideal biomarker for many diseases [100,101]. Serum and urinary miRNAs were shown to be associated with different disease characteristics of SLE [102]. Another study demonstrated that circulating miRNAs were found systematically altered in SLE, and a 4-miRNA signature could be used to discriminate SLE with healthy controls, with another set of miRNA associated with lupus nephritis [103]. With the development of the detection technique for circulating miRNA, new technologies used for profiling miRNA expression and more expression data obtained, miRNA will possibly become a convenient biomarker choice for SLE and its associated organ damage [97,104].

## 7. The Therapeutic Perspective of miRNAs in SLE

Besides the basic research on the mechanisms for the pathogenesis of SLE, there are many research works focused on how to translate the knowledge of miRNAs in SLE into the development of novel therapies. Recent studies revealed that the active metabolite of mycophenolate mofetil (MMF), which is widely used for the treatment of SLE, could upregulate the expression of *miR-142-3p/5p* and *miR-146a* in T-cells [105]. This finding suggested that the therapeutic effects of MMF might partially be mediated by these two miRNAs and that we could develop more specific therapeutic methods by using these two miRNAs. Since we have known the mechanisms of certain miRNAs regulating the abnormal activation of the immune responses in SLE, there have been many efforts made to explore the possibility of using miRNA as therapeutic targets or treatment reagents for SLE. *miR-155* has long been established as a regulator of B-cell functions [106,107]. Based on these findings, *miR-155* might take part in regulating the production of autoantibodies in SLE. In fact, ablation of *miR-155* in MRL-lpr lupus-prone mice reduced autoantibody production with the alleviation of kidney inflammation [108]. Another research work demonstrated the deficiency of *miR-155* in mice protected them from Pristane-induced lupus-associated pulmonary hemorrhage and application of a synthetic *miR-155* antagomir in the mice could ameliorate pulmonary hemorrhage induced by Pristane [109]. Recent studies showed that overexpression of *miR-155* could inhibit the expression of PP2Ac, which negatively regulates the expression of *IL2* in PBMCs. Thus, the overexpression of *miR-155* could possible relieve the inhibition of *IL2* by PP2Ac in juvenile SLE disease [110]. These results indicate that blocking *miR-155* might benefit SLE patients. Further, *in vivo* silencing of *miR-21* by seed-targeting LNA altered the ratios of CD4/CD8 T-cell-reversed splenomegaly and reduced the number of Fas receptor-expressing lymphocytes [111]. Another promising miRNA is *miR-146a*, which is recognized as a major negative regulator of immune response, and its deficiency led to multiple immune disorders [53,112,113]. Consistent with previous findings of *miR-146a*’s regulatory functions in immune response, injection of *miR-146a* agomirs, a chemical-modified *miR-146a* mimic, rendered the mice resistant to Pristane-induced hemorrhagic pulmonary capillaritis with suppressed interferon response [114]. As an example of miRNA-based therapy, inhibitors of *miR-122* are used in clinical trials to test their activity in HCV (hepatitis C virus) infection [115]. Although there are still issues that need to be solved, such as developing efficient delivery approaches and chemical modifications for the improvement of the absorption and the stability of synthetic oligonucleotides, as well as testing the pharmacokinetic traits and side effects of administrating miRNA mimics or inhibitors *in vivo*, utilizing miRNAs as therapeutic molecules is still a promising choice to treat SLE patients in the future [115]. (Table 1 summarizes the targets and the signaling pathways of the miRNAs in SLE discussed above).

**Table 1:** miRNAs in the pathogenesis of systemic lupus erythematosus (SLE).

miRNA	Target	Regulated Process	References
let-7a	IL6	IL6 induction	[65]
let-7c	Blimp1, SOCS1	Activation of DCs	[64]
miR-125a	KLF13	CCL5 induction in T-cells	[81]
miR-126	DNMT1	DNA methylation in T-cells	[73]
miR-146a	TRAF6, IRAK1	NFκB mediated inflammatory response	[53]
	TRAF6, IRAK1, IRAK2	RIG-I-dependent anti-viral pathway	[54]
	IRF5, STAT1	Type I IFN induction and signaling	[55]
miR-148a	DNMT1	DNA methylation in T-cells	[71]
miR-150	SOCS1	Renal fibrosis	[94]
miR-155	MyD88, TAB2	TLR/IL1 inflammatory pathway	[57,58]
	SOCS1	Type I IFN signaling	[59]
	PP2Ac	IL2 induction	[110]
miR-155*	IRAKM	Type I IFN induction	[ 60]
miR-17~92	PTEN, Bim	Proliferation of lymphocytes	[84]
	Rora, PHLPP2	Differentiation and function of Tfh cells	[85,86]
miR-21	RASGRP1	DNA methylation in T-cells	[71]
miR-23b	TAB2, TAB3, IKKα	IL17, TNFα, IL1β signaling	[91]
miR-29b	Sp1	DNA methylation in T-cells	[74]
miR-30a	Lyn	Activation of B-cells	[87]
miR-31	RhoA	IL2 induction in T-cells	[80]
miR-3148	TLR7	TLR7 inflammatory pathway	[63]

## 8. Conclusions

As we are still at the early stage of the exploration of miRNA’s essential roles in SLE pathogenesis, there are many unresolved questions. The application of animal models will advance the field by facilitating the study of the *in vivo* function of miRNAs in SLE. Profiling the expression of miRNAs still needs to be done to obtain more precise expression patterns of miRNAs among SLE patients with different ages, or different races, or different genders, or different disease manifestations and to identify novel specific biomarkers. The identification of new targets of miRNAs will give us a more comprehensive view of the mechanisms of miRNAs in regulating immune responses in SLE. Developing new techniques to precisely and conveniently measure the expression of miRNAs in our body fluid and novel methods to efficiently overexpress or inhibit the activities of miRNAs *in vivo* will further accelerate the translational application of miRNAs as novel diagnosis and therapeutic methods in SLE.
